# 苯达莫司汀联合抗CD20单抗一线治疗老年惰性B细胞非霍奇金淋巴瘤的多中心回顾性研究

**DOI:** 10.3760/cma.j.cn121090-20241227-00597

**Published:** 2025-09

**Authors:** 姝超 秦, 祎 缪, 昭亮 张, 捷 张, 玉叶 史, 雨青 苗, 伟英 顾, 维成 郑, 祝霞 贾, 国强 林, 海雯 倪, 小红 徐, 敏 徐, 晓艳 谢, 铃 王, 芸 庄, 巍 张, 萍 柳, 建勇 李, 文瑜 施

**Affiliations:** 1 南京医科大学第一附属医院，江苏省人民医院血液科，南京 210029 Department of Hematology, the First Affiliated Hospital of Nanjing Medical University, Jiangsu Province Hospital, Nanjing 210029, China; 2 南通大学附属医院肿瘤科，南通 226001 Department of Oncology, Affiliated Hospital of Nantong University, Nantong 226001, China; 3 淮安市第一人民医院血液科，淮安 223000 Department of Hematology, Huai'an First People's Hospital, Huai'an 223000, China; 4 盐城市第一人民医院血液科，盐城 224001 Department of Hematology, the First People's Hospital of Yancheng, Yancheng 224001, China; 5 常州市第一人民医院血液科，常州 213003 Department of Hematology, the First People's Hospital of Changzhou, Changzhou 213003, China; 6 苏州大学附属第二医院血液科，苏州 215000 Department of Hematology, the Second Affiliated Hospital of Soochow University, Suzhou 215000, China; 7 南京医科大学附属常州市第二人民医院血液科，常州 213003 Department of Hematology, Affiliated Changzhou Second Hospital of Nanjing Medical University, Changzhou 213003, China; 8 徐州医科大学附属淮安医院，淮安市第二人民医院血液科，淮安 223002 Department of Hematology, Affiliated Huai'an Hospital of Xuzhou Medical University, Huai'an Second People's Hospital, Huai'an 223002, China; 9 江苏省中医院血液科，南京 210029 Department of Hematology, Jiangsu Province Hospital of Chinese Medicine, Nanjing 210029, China; 10 南通市肿瘤医院血液淋巴瘤科，南通 226361 Department of Hematology and Lymphoma, Nantong Tumor Hospital, Nantong 226361, China; 11 张家港市第一人民医院血液科，张家港 215600 Department of Hematology, the General Public Hospital of Zhangjiagang, Zhangjiagang 215600, China; 12 江苏省苏北人民医院血液科，扬州 225009 Department of Hematology, Northern Jiangsu People's Hospital Affiliated to Yangzhou University, Yangzhou 225009, China; 13 南通市第一人民医院血液科，南通 226014 Department of Hematology, Nantong First People's Hospital, Nantong 226014, China; 14 无锡市人民医院血液科，无锡 214023 Department of Hematology, Wuxi People's Hospital, Wuxi 214023, China; 15 南京市江宁医院，南京医科大学附属江宁医院血液科，南京 211100 Department of Hematology, the Affiliated Jiangning Hospital of Nanjing Medical University, Nanjing 211100, China; 16 无锡市第二人民医院血液科，无锡 214000 Department of Hematology, Wuxi No.2 People's Hospital, Wuxi 214000, China

**Keywords:** 盐酸苯达莫司汀, 老年, 惰性B细胞性淋巴瘤, 一线治疗, Bendamustine hydrochloride, Elderly, B-cell indolent lymphoma, First-line therapy

## Abstract

**目的:**

评估苯达莫司汀联合抗CD20单抗一线治疗老年惰性B细胞非霍奇金淋巴瘤（B-iNHL）患者的疗效与安全性。

**方法:**

回顾性分析2019年12月1日至2024年4月20日期间在江苏省淋巴瘤协作组16家医院就诊的159例老年B-iNHL患者应用苯达莫司汀联合抗CD20单抗（利妥昔单抗或奥妥珠单抗）治疗的疗效及安全性。其中139例（87.4％）应用BR（苯达莫司汀+利妥昔单抗）方案治疗，20例（12.6％）应用BG（苯达莫司汀+奥妥珠单抗）方案治疗。

**结果:**

159例患者中男101例（63.5％）、女58例（36.5％），中位年龄为69（60～84）岁。138例（86.8％）患者可评估疗效，其中75例（54.3％）患者达完全缓解，52例（37.7％）患者达部分缓解，总有效率为92.0％。中位随访24（4～64）个月，患者无进展生存率为（87.5±3.0）％，总生存率为（83.2±3.3）％。死亡的27例患者中，仅6例（22.2％）伴有疾病进展。每周期苯达莫司汀中位应用剂量为73.0（50.8～89.7）mg·m^−2^·d^−1^，第1、2天用药。53例（33.3％）患者出现3级及以上的不良反应，感染（30例，18.9％）发生率最高，其次是中性粒细胞减少（24例，15.1％）。

**结论:**

真实世界老年B-iNHL患者一线应用苯达莫司汀联合抗CD20单抗疗效较好，安全性可控，耐受性较好。

## Abstract (en)

**Objective:**

To investigate the efficacy and safety of bendamustine combined with anti-CD20 monoclonal antibody in the first-line treatment of older patients with indolent B-cell non-Hodgkin lymphoma（B-iNHL）.

**Methods:**

The clinical data of 159 patients with B-iNHL enrolled in 16 hospitals from Jiangsu Cooperative Lymphoma Group from December 1, 2019, to April 20, 2024, were analyzed for regimen efficacy and safety. Bendamustine plus rituximab（BR）and bendamustine plus obinutuzumab（BG）were administered to 139（87.4％）and 20（12.6％）patients, respectively.

**Results:**

Among the 159 patients, 101（63.5％）were male and 58（36.5％）were female, with a median age of 69 years（range: 60–84）. Efficacy could be assessed in 138（86.8％）patients. The efficacy assessment demonstrated that the overall response rate was 92.0％ with complete and partial remissions in 75（54.3％）and 52（37.7％）cases, respectively. With a median follow-up of 24 months（range: 4–64）, the progression-free survival rate was（87.5±3.0）％ and the overall survival rate was（83.2±3.3）％. Of the 27 patients who died, 6（22.2％）died due to disease progression. The mean applied dose of bendamustine per cycle was 73.0（50.8–89.7）mg/m² per day, administered on days 1 and 2. Adverse events of grade 3 or higher were reported in 53（33.3％）patients, with infection（30 cases,18.9％）and neutropenia（24 cases, 15.1％）demonstrating the highest incidence.

**Conclusion:**

Bendamustine combined with anti-CD20 monoclonal antibody demonstrated good efficacy and is well-tolerated in the first-line treatment of elderly patients with B-iNHL.

苯达莫司汀是一种兼具烷化剂和嘌呤类似物双重功能的细胞毒性药物，广泛应用于B细胞性惰性非霍奇金淋巴瘤（B-iNHL）的治疗。StiL研究和BRIGHT研究均显示BR（苯达莫司汀+利妥昔单抗）方案在B-iNHL患者中疗效佳、耐受性良好，奠定了其在B-iNHL中的一线治疗地位[1]–[2]。苯达莫司汀联合抗CD20单抗方案目前已获国内外多项指南或共识一线推荐[3]–[6]。B-iNHL中位发病年龄为65～70岁，年龄是预后的独立危险因素[7]–[8]，老年患者预后差、对治疗耐受性不佳。本研究回顾性分析苯达莫司汀联合抗CD20单抗一线治疗老年B-iNHL患者的疗效与安全性相关的数据。

## 病例与方法

1. 病例：本研究纳入自2019年12月1日至2024年4月20日在江苏省淋巴瘤协作组16家医院就诊的159例老年B-iNHL患者，包括滤泡性淋巴瘤（FL）、边缘区淋巴瘤（MZL）、淋巴浆细胞淋巴瘤/华氏巨球蛋白血症（LPL/WM）、慢性淋巴细胞白血病/小淋巴细胞淋巴瘤（CLL/SLL）及部分B细胞慢性淋巴增殖性疾病不能分类型（B-CLPD-U）。入组患者一线均接受苯达莫司汀联合抗CD20单抗（利妥昔单抗或奥妥珠单抗）治疗。收集患者的临床资料，包括性别、年龄、病理类型、疾病分期、有无大包块（长径≥7 cm）、有无骨髓浸润及美国东部肿瘤协作组（ECOG）体能状态（PS）评分等。

纳入标准包括：①年龄≥60岁；②所有患者既往均未接受过治疗；③所有患者均在治疗前进行组织活检，病理诊断均符合世界卫生组织（WHO）2016年淋巴组织肿瘤分类标准[9]；④CLL/SLL患者体能状态良好并伴有免疫球蛋白重链可变区（IGHV）基因突变，无del（17p）/TP53基因突变。不同类型淋巴瘤的治疗指征参考我国淋巴瘤诊断与治疗指南[3],[5],[10]。CLL患者的临床分期采用Binet和Rai分期。除LPL/WM和CLL患者外，其余患者的临床分期采用2014年版Lugano分期标准[11]–[12]。

2. 治疗方案：159例患者中，139例（87.4％）应用BR方案治疗（利妥昔单抗375 mg/m^2^第0天；苯达莫司汀70～90 mg·m^−2^·d^−1^第1～2天），共6个周期；20例（12.6％）应用BG方案治疗［奥妥珠单抗1 000 mg第0天（第1个周期于第0、7、14天分别给予1 000 mg，其中CLL患者首次应用第0天100 mg，第1天900 mg，以提高输注安全性）；苯达莫司汀70～90 mg·m^−2^·d^−1^第1～2天］，共6个周期。FL患者中28例（70.0％）应用BR方案，12例（30.0％）应用BG方案治疗；MZL患者中46例（88.5％）应用BR方案，6例（11.5％）应用BG方案治疗。47例（29.6％）患者后续接受了抗CD20单抗的维持治疗，其中38例应用BR方案治疗的患者接受利妥昔单抗维持治疗，9例应用BG方案治疗的患者接受奥妥珠单抗维持治疗。苯达莫司汀首次用药剂量根据患者体能状况调整，治疗期间由临床医师判定不良反应与药物的相关性并依据药品说明进行剂量调整。

3. 疗效评估：治疗前收集患者基线数据，3个周期治疗（中期评估）以及6个周期治疗结束后对患者进行疗效评估。CLL/SLL的疗效评估依据《中国慢性淋巴细胞白血病/小淋巴细胞淋巴瘤的诊断与治疗指南（2022年版）》[5]，LPL/WM的疗效评估依据《淋巴浆细胞淋巴瘤/华氏巨球蛋白血症诊断与治疗中国指南（2022年版）》[3]，主要缓解率（MRR）定义为至少获得部分缓解（PR）的患者比例。其余患者的疗效评估依据2014年版Lugano标准[11]–[12]，评估总缓解率（ORR）、完全缓解（CR）率、PR率、疾病稳定（SD）及疾病进展（PD）率。ORR为CR率和PR率之和。

4. 安全性评价：不良事件（AE）根据常见不良反应事件评价标准（CTCAE）5.0版进行分级。

5. 随访：随访信息来自住院和门诊病历以及电话随访记录。随访截止时间为2024年12月20日。无进展生存（PFS）期定义为从疾病确诊开始到PD或因任何原因死亡的时间；总生存（OS）期定义为从疾病确诊开始至因任何原因死亡或最后一次随访的时间。

6. 统计学处理：应用SPSS 26.0软件和Graphpad prism 9.0软件进行统计学分析，计数资料采用例数（％）描述，计量资料采用*M*（范围）或*x*±*s*描述，采用Kaplan-Meier法描绘生存曲线，采用卡方检验或Fisher精确检验法进行组间比较，双侧*P*<0.05为差异具有统计学意义。

## 结果

1. 临床特征：159例患者中男101例（63.5％）、女58例（36.5％），中位年龄为69（60～84）岁。其中MZL 52例（32.7％）、FL 40例（25.2％）、LPL/WM 26例（16.4％）、CLL/SLL 25例（15.7％）、B-CLPD-U 16例（10.1％）。CLL患者中Binet C期15例（60.0％），其余患者中Ann Arbor分期Ⅲ期15例（13.9％）、Ⅳ期71例（65.7％）。骨髓受累98例（61.6％）。淋巴结直径≥7 cm 21例（13.2％）。可评估的135例患者中，ECOG PS评分≥2分39例（28.9％）。

2. 苯达莫司汀用量及疗效：苯达莫司汀中位应用剂量为73.0（50.8～89.7）mg·m^−2^·d^−1^第1～2天，中位治疗6（1～6）个周期。159例患者中138例（86.8％）患者可评估疗效。6个周期治疗结束后，75例（54.3％）患者达CR，52例（37.7％）患者达PR，ORR为92.0％。其中LPL/WM患者的MRR为92.3％，其余各病种的疗效见表1。

**表1:** 苯达莫司汀联合抗CD20单抗一线治疗老年B细胞性惰性非霍奇金淋巴瘤患者疗效分析

疗效	总体（138例）	MZL（47例）	FL（36例）	LPL/WM（26例）	CLL/SLL（23例）	B-CLPD-U（6例）
ORR（％）	92.0	95.8	94.4	92.3	87.0	66.7
CR［例（％）］	75（54.3）	31（66.0）	26（72.2）	7（26.9）	9（39.1）	2（33.3）
PR［例（％）］	52（37.7）	14（29.8）	8（22.2）	17（65.4）	11（47.8）	2（33.3）
SD［例（％）］	2（1.4）	1（2.1）	0（0）	0（0）	0（0）	1（16.7）
PD［例（％）］	9（6.5）	1（2.1）	2（5.6）	2（7.7）	3（13.0）	1（16.7）

**注** MZL：边缘区淋巴瘤；FL：滤泡性淋巴瘤；LPL/WM：淋巴浆细胞淋巴瘤/华氏巨球蛋白血症；CLL/SLL：慢性淋巴细胞白血病/小淋巴细胞淋巴瘤；B-CLPD-U：B细胞慢性淋巴增殖性疾病不能分类型；ORR：总缓解率；CR：完全缓解；PR：部分缓解；SD：疾病稳定；PD：疾病进展

中位随访时间为24（4～64）个月，中位PFS期及OS期均未达到。患者总体2年PFS率为（87.5±3.0）％、OS率为（83.2±3.3）％。其中MZL的PFS率为（90.0±4.8）％，FL的PFS率为（85.5±6.1）％，LPL/WM的PFS率为（85.7±7.7）％，CLL/SLL的PFS率为（89.1±7.5）％。

亚组分析显示，在MZL患者及FL患者中，应用BR方案及BG方案的患者PFS及OS差异均无统计学意义［2年PFS率：MZL，（91.4±4.8）％对（83.3±15.2）％，*P*＝0.544；FL，（83.2±7.8）％对（90.9±8.7）％，*P*＝0.394；2年OS率：MZL，（92.0±4.4）％对（80.0±17.9）％，*P*＝0.342；FL，（84.1±7.3）％对（88.9±8.1）％，*P*＝0.348）。MZL患者中16例（31.4％）接受了抗CD20单抗维持治疗，其PFS优于未接受抗CD20单抗维持治疗患者（*P*＝0.045），OS差异无统计学意义（*P*＝0.104）（图1）。FL患者中24例（61.5％）接受了抗CD20单抗维持治疗，其PFS优于未接受抗CD20单抗维持治疗患者（*P*＝0.041），OS差异无统计学意义（*P*＝0.452）（图2）。苯达莫司汀应用剂量≥73.0 mg·m^−2^·d^−1^的患者与<73.0 mg·m^−2^·d^−1^的患者PFS与OS差异均无统计学意义［2年PFS率：（85.9±5.4）％对（81.1±6.5）％，*P*＝0.352；2年OS率：（88.7±4.8）％对（85.6±6.7）％，*P*＝0.872］。

**图1:**
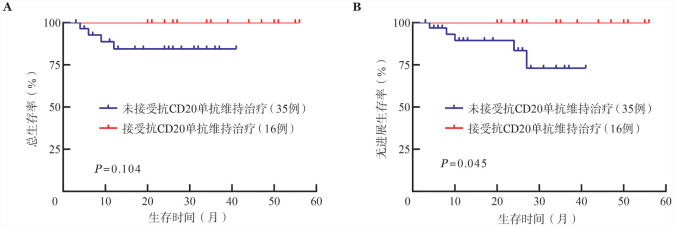
边缘区淋巴瘤患者基于是否接受抗CD20单抗维持治疗分组的总生存（A）及无进展生存（B）曲线

**图2:**
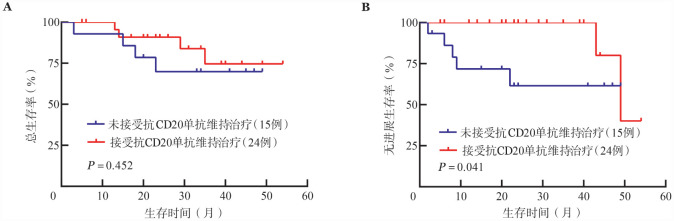
滤泡性淋巴瘤患者基于是否接受抗CD20单抗维持治疗分组的总生存（A）及无进展生存（B）曲线

3. 安全性：53例（33.3％）患者出现3级及以上的AE，其中感染（30例，18.9％）发生率最高，其次是中性粒细胞减少（24例，15.1％）。5例（3.1％）因治疗相关的AE进行苯达莫司汀减量，6例（3.8％）无法耐受后续治疗而停药，1例患者在1个周期用药后发生心源性猝死。出现3级及以上感染的患者中，肺部感染27例（17.0％），新型冠状病毒感染14例（8.8％），败血症2例（1.2％），泌尿系感染1例（0.6％）。亚组分析显示，苯达莫司汀应用剂量≥73.0 mg·m^−2^·d^−1^的患者发生3级及以上AE（53.2％对22.2％，*P*＝0.002）、中性粒细胞减少（25.5％对6.7％，*P*＝0.014）的比例高于应用剂量<73.0 mg·m^−2^·d^−1^的患者。接受抗CD20单抗维持的患者发生3级及以上AE（45.8％对27.8％，*P*＝0.014）、3级及以上感染（29.2％对13.9％，*P*＝0.013）的比例高于未接受抗CD20单抗维持的患者。其余3级及以上AE包括血小板减少（6例，3.8％）、贫血（2例，1.2％）、皮疹（4例，2.5％）、肝功能异常（2例，1.2％）。

死亡的27例患者中，仅6例（22.2％）伴有疾病进展。其余已知死亡原因的13例患者中，5例死于肺部感染导致的呼吸衰竭、3例死于心跳骤停、3例死于第二肿瘤（胃癌、肝癌及阑尾黏液性肿瘤各1例）、2例死于脑卒中。

## 讨论

B-iNHL是临床常见的淋巴瘤类型，患者发病年龄高，发病率随年龄增加而上升，随着我国人口老龄化进程加速，老年B-iNHL患者的发病率和占比逐年增加，对该群体的治疗和管理已成为亟待解决的临床挑战。FL国际预后指数2（FLIPI-2）、CLL国际预后指数（CLL-IPI）、WM国际预后评分系统（IPSS WM）、黏膜相关淋巴组织淋巴瘤国际预后指数（MALT-IPI）均将年龄纳入预后独立危险因素。老年B-iNHL患者不仅人数逐年增加，且通常伴随预后较差和治疗耐受性较低的问题，显著影响其疾病管理和生存结局。现行指南建议老年患者采用包括BR方案在内的一线治疗[5]。StiL研究和BRIGHT研究均显示BR方案在iNHL中的疗效和安全性优于传统的R-CHOP（利妥昔单抗+环磷酰胺+多柔比星+长春新碱+泼尼松）方案[1]–[2]。BRISMA/IELSG36研究提示BR方案在脾边缘区淋巴瘤（SMZL）患者中安全有效，入组患者中位年龄66岁，73％的患者大于60岁[13]，2024年该研究的更新数据显示，接受BR方案治疗的SMZL患者微小残留病（MRD）阴性率高，6个周期治疗结束后54％的患者MRD阴性，缓解深度良好[14]。

本研究回顾性分析了多中心一线应用苯达莫司汀联合抗CD20单抗治疗老年B-iNHL患者的临床数据，考虑长期口服布鲁顿酪氨酸激酶抑制剂（BTKi）带来的经济压力，部分伴有IGHV基因突变、不伴del（17p）或TP53基因突变CLL/SLL患者选择接受BR或BG方案，未使用BTKi治疗，纳入本研究队列。本研究CR率达到54.3％，高于StiL和BRIGHT研究（分别为40％和31％）。在MZL亚组中，CR率为66.0％、PR率为29.8％、ORR达到95.8％，与BRISMA/IELSG36研究（CR率73％、PR率18％、ORR 91％）结果相近，进一步支持了该方案在MZL患者中的应用价值[13]。国内两项研究[15]–[16]应用BR方案一线治疗iNHL和MCL患者，中位年龄均为55岁，ORR分别为98.6％及95.2％，与本研究结果相似；CR率分别为83.3％及77.8％，高于本研究CR率，可能与本研究患者中位年龄高、骨髓受累比例高等因素相关。本研究进一步在FL和MZL亚组中比较了BG和BR方案疗效，发现在中位随访2年时，两种方案PFS和OS差异无统计学意义，可能与本研究随访时间较短、入组老年患者等因素相关。

Arulogun等[17]研究显示，在一线接受BR方案治疗的WM患者群体中，苯达莫司汀用药剂量影响一线和复发患者的缓解和生存结局。本研究老年患者总体接受苯达莫司汀治疗的剂量较低，每周期苯达莫司汀中位应用剂量为73.0 mg·m^−2^·d^−1^，低于国外临床试验[1]–[2]。其中WM患者每周期苯达莫司汀中位应用剂量为70.3 mg·m^−2^·d^−1^，按照用药总剂量换算，相当于Arulogun等[17]的研究中800～999 mg/m^2^剂量组，本研究WM患者MRR为92.3％，疗效优于该剂量组（MRR为81.8％）。针对苯达莫司汀不同剂量组疗效进一步分析显示，每周期苯达莫司汀应用剂量≥73.0 mg·m^−2^·d^−1^的患者与<73.0 mg·m^−2^·d^−1^的患者PFS与OS差异均无统计学意义。

PRIMA研究[18]显示，与观察组相比，一线治疗后利妥昔单抗维持治疗显著改善了FL患者的PFS（中位PFS期：10.5年对4.1年，*P*<0.001），但PFS的延长并未转化成OS的获益[18]。维持组3～4级AE和严重AE发生率较观察组高（24.4％和16.9％，21.2％和13.4％）[18]。本研究在MZL和FL亚组中分析了抗CD20单抗维持的疗效和安全性，发现接受抗CD20单抗维持治疗的患者取得了PFS获益，但与未接受抗CD20单抗维持治疗的患者相比，发生3级及以上感染的比例显著增高。

苯达莫司汀最显著的治疗相关不良反应包括中性粒细胞减少及感染。Manos等[19]的回顾性研究报道，44％的患者发生感染，3级及以上感染占比达36％。年龄≥60岁与感染事件增加相关。接受单纯疱疹病毒（HSV）/水痘-带状疱疹病毒（VZV）预防与耶氏肺孢子菌（PJP）预防的患者，感染的发生率显著降低[19]。Shotton等[20]针对苯达莫司汀治疗NHL安全性的研究显示，接受了苯达莫司汀联合或不联合利妥昔单抗治疗的患者中，48％发生了3级及以上AE，24％发生了3级及以上感染，13％因无法耐受AE而停药。单因素分析提示年龄与不良反应发生率无相关性[20]。StiL、BRIGHT和GALLIUM研究也显示苯达莫司汀联合抗CD20单抗方案感染发生率较高[1]–[2],[21]。此外，多项回顾性研究提示在CAR-T细胞采集前应用苯达莫司汀会对CAR-T细胞的制造及安全性产生负面影响[22]–[23]。本研究老年患者3级及以上AE的发生率及因无法耐受AE而停药的患者比例均低于既往研究的报告数据。其中基于用药剂量的亚组分析显示，每周期苯达莫司汀应用剂量≥73.0 mg·m^−2^·d^−1^的患者发生3级及以上AE、中性粒细胞减少的比例高于应用剂量<73.0 mg·m^−2^·d^−1^的患者。患者总体耐受性良好，AE发生率低于国外数据，考虑与苯达莫司汀应用剂量较低、应用生长因子支持以及部分研究中心从患者接受治疗开始至免疫恢复前采取抗HSV/VZV和PJP预防有关。

综上所述，本研究表明，在真实世界老年B-iNHL患者中，苯达莫司汀联合抗CD20单抗一线治疗方案具有良好的疗效和可控的安全性。患者安全性较高可能与以下因素密切相关：苯达莫司汀的减低剂量应用、生长因子支持以及HSV/VZV和PJP的有效预防措施。本研究为专门针对老年B-iNHL患者的多中心研究，样本量大、随访时间长，比较了应用不同剂量苯达莫司汀的疗效及安全性数据。在抗CD20单抗的应用上，除联合利妥昔单抗外还初步比较了联合奥妥珠单抗以及是否接受抗CD20单抗维持治疗的疗效和安全性数据。本研究为回顾性设计，存在一定局限性。因此，需进一步开展大样本量的前瞻性研究以全面评估该治疗方案的长期疗效和安全性。
